# In Silico Predictions Driving the Development of 3D-Printed Drug Delivery Systems

**DOI:** 10.3390/pharmaceutics18010032

**Published:** 2025-12-26

**Authors:** Pooja Todke, Robertas Lazauskas, Jurga Bernatoniene

**Affiliations:** 1 Institute of Pharmaceutical Technologies, Faculty of Pharmacy, Medical Academy, Lithuanian University of Health Sciences, 44307 Kaunas, Lithuania; pooja.ashok.todke@lsmu.lt; 2 Department of Drug Technology and Social Pharmacy, Faculty of Pharmacy, Medical Academy, Lithuanian University of Health Sciences, 44307 Kaunas, Lithuania; 3 Institute of Physiology and Pharmacology, Medical Academy, Lithuanian University of Health Sciences, 50161 Kaunas, Lithuania; robertas.lazauskas@lsmuni.lt

**Keywords:** in silico screening, 3D printed drug delivery systems, predictability, printability, dissolution

## Abstract

**Background:** Three-dimensional printing (3DP) is a promising technology for advancing pharmaceutical research by enabling the production of personalized drug products. However, its progress has been hindered by the conventional trial-and-error approach to excipient selection and optimization. **Methods:** In this study, the blend module was employed to determine the miscibility parameters—mixing energy (E_mix_) and Flory–Huggins interaction parameter (χ) to find the right excipients and drug–excipient ratio and examine the incorporation of plasticizers and lipids to enhance printability. Furthermore, molecular dynamics (MD) simulations were employed to calculate the cohesive energy density (CED) for predicting the dissolution behavior of 3DP formulations. **Results:** Data from 51 formulations were analyzed, enabling correlation and experimental validation of the in silico predictions. The predicted miscibility values demonstrated a strong correlation with experimental printability results. Furthermore, using a miscibility parameter, it was possible to accurately forecast minor changes in the drug-to-excipient ratio, plasticizer/lipid concentration, and hot-melt extrusion (HME) temperature that affect printability. Hydrophilic carriers exhibited lower CED values corresponding to faster drug release. In contrast, more hydrophobic carriers revealed high CED values, indicating stronger drug entrapment and sustained release. **Conclusions:** The miscibility parameters and MD-simulated CED values provide a practical framework for early-stage, high-throughput excipient screening. Overall, in silico prediction offers a viable strategy for modeling the entire 3DP workflow, minimizing the need for trial-and-error experimentation, and thereby accelerating the clinical translation of 3DP drug delivery systems.

## 1. Introduction

The advent of three-dimensional printing (3DP) has introduced a significant shift in healthcare, opening new avenues for tailored drug delivery systems (DDS). This technology enables precise modulation of drug release kinetics, thereby enhancing therapeutic efficacy and improving patient adherence [1,2,3]. It also facilitates the combination of multiple drugs within a single dosage form, simplifying complex treatment regimens. With its rapid prototyping and on-demand manufacturing capabilities, 3DP significantly shortens development timelines while lowering production costs. Unlike conventional tablet manufacturing, 3DP offers transformative advantages, including single-step fabrication, individualized dose customization, and the creation of sophisticated polypill combinations through additive manufacturing [4,5]. Moreover, innovations such as multi-material printing and four-dimensional (4D) printing further extend its potential by integrating diverse functional materials, opening vast opportunities for the future of personalized DDS [6].

However, widespread pharmaceutical adoption faces significant technical challenges, especially related to material properties. Effective 3DP implementation requires excipients that are carefully balanced to ensure chemical and physical compatibility between the API and excipients, as this is critical to avoid degradation and instability or reduced therapeutic performance [7,8,9]. In this context, compatibility refers to the miscibility between the drug and polymer or other excipients, determining whether the drug can remain molecularly dispersed without crystallizing during processing. To achieve both extrudability for filaments and printability for dosage forms, it is essential that the selected excipients are molecularly miscible with the API or with other formulation components. Such miscibility ensures uniform dispersion, stable structure, and consistent performance of the final printed dosage forms [10,11,12,13].

Printability has been defined as the ability of a formulation or material to be processed by a 3DP technique to produce structures with consistent geometry, dimensional accuracy, and mechanical stability [14]. It depends on process-specific requirements, such as melt viscosity and mechanical strength for fused deposition modeling (FDM), photopolymerization behavior for stereolithography (SLA), and powder flow or sintering characteristics for selective laser sintering (SLS). Printability is also strongly influenced by drug–excipient miscibility, which governs melt homogeneity, rheology, and overall material performance during printing [15,16].

MD simulations, particularly nonequilibrium, have been widely used to predict rheological properties and viscosity by applying shear through momentum-flux or source–sink methods, enabling the capture of non-Newtonian behaviors such as shear thinning in polymeric systems relevant to pharmaceutical 3DP [17,18]. Recent studies have shown that rheological parameters can reliably predict both printability and dissolution performance for FDM-based formulations. For instance, semi-empirical models have identified optimal filament viscosity ranges for successful extrusion, while machine-learning-based models have further extended these predictions to dissolution behavior using rheological inputs [19]. Machine learning has also significantly accelerated formulation design of 3D-DDS. For example, the M3DISEEN platform predicted filament properties and printability from 614 formulations. Similarly, a large-scale study using 1594 FDM/HME formulations successfully predicted printability, filament characteristics, processing temperatures, and drug release profiles [20]. In addition, multimodal machine-learning models have been developed to accurately predict the printability of SLS-based drug products [21]. However, current machine-learning methods remain largely dependent on literature-based formulation databases, which limit their applicability for new 3DP developments [22,23].

Other computational techniques, such as computational fluid dynamics, enable prediction of melt flow, pressure distribution, shear rate, and temperature evolution inside the nozzle or extrusion zone, helping to identify conditions that minimize clogging, ensure uniform filament formation, and maintain drug stability during printing [24]. Finite-element analysis is employed to evaluate the mechanical performance of printed dosage forms, including stress distribution, layer adhesion, deformation, and structural integrity during compression, handling, or swelling during dissolution [25].

However, even when suitable excipients are identified, predicting their performance remains challenging. Recent studies employed Hansen solubility parameters (HSPs) for screening polymeric carriers, cosolvent systems, and 3D-printed pharmaceutical filaments, correlating the HSP distances with drug loading, dissolution, and stability [26]. Advanced computational models, such as COSMO-RS, further enhance HSP predictions for complex molecules, streamlining excipient selection and drug solubilization strategies in drug delivery development [27]. Nonetheless, HSPs have key limitations, including the lack of explicit temperature dependence due to solubility spheres being treated as constant, approximate mixture rules that fail in non-ideal solvent blends, and, for novel excipients, suboptimal scoring, theoretical constraints, and limited data that can reduce predictive accuracy [28,29,30,31]. Likewise, Kirkwood–Buff theory (KBF) predicts drug–excipient interactions and miscibility by linking molecular pair correlations to thermodynamic properties like preferential solvation and activity coefficients, offering a powerful tool for formulation design [32]. Also, KBF integrates it with MD and miscibility parameters to model API solubility in cosolvents and polymer matrices, revealing composition-dependent χ parameters and phase diagrams for systems like local anesthetics with hydrotropes [33]. These advances improved predictions of amorphous solid dispersion stability and dissolution behavior, addressing convergence issues in simulations for accurate rheology and printability assessments [34]. Among in silico approaches, excipient screening is one of the most extensively studied strategies [35,36,37]. Nevertheless, main challenges still exist, including selecting suitable excipients, determining optimum drug-to-excipient ratios, and reliably predicting printability and dissolution profiles.

To address these challenges, the present study reports a comprehensive in silico investigation utilizing the literature’s 3DP formulation, and the Flory–Huggins (FH) interaction parameter (χ) or mixing energy (E_mix_) miscibility parameters are crucial for predicting printability. A negative χ and E_mix_ values generally indicate better miscibility between the drug and excipients, leading to a more homogeneous and printable formulation. Previous studies have applied the FH theory to evaluate API–excipient miscibility and its impact on drug loading in amorphous solid dispersions [38]. Ruiz-Cantu et al. applied drop-on-demand inkjet 3DP combined with high-throughput screening and an FH miscibility model to predict microstructures arising from phase separation. Their findings revealed that the release performance of 3DP formulations can be inferred from the behavior of single drops [39].

Cohesive energy density (CED) can be used to predict the dissolution behavior of materials. The CED is defined as the tendency of a solid to dissolve in a solvent [40]. The CED was derived from the intermolecular non-bonded energy after eliminating the intramolecular bonded contributions from the total non-bonded energy. By estimating and comparing CED values, it becomes possible to forecast how readily a solid material will dissolve in a given solvent, facilitating the prediction of dissolution performance based on molecular interaction characteristics [41]. Previous studies have employed CED analysis to investigate excipient miscibility, showing that CED decreases with increasing crosslink density and temperature, with a more pronounced temperature effect observed in polymers with a lower conversion degree [42]. CED has also been employed to study the cohesion of polymer mixtures, showing a strong dependence on both temperature and polymer concentration, which is crucial for understanding viscoelastic performance in ophthalmic formulations [43]. Collectively, these in silico parameters can contribute to the rapid and accurate development of 3D-printed formulations.

The present study aims to conduct a comprehensive in silico investigation for the rational design of 3D-printed drug delivery systems (3DP-DDS) using a high-throughput screening approach. The objectives are as follows: (1) Printability prediction—assessing the miscibility of drug–excipient systems, drawing on prior formulations where computational methods have been employed to predict the printability of 3DP-DDS; and (2) dissolution prediction—applying predictive molecular dynamics (MD) tools to estimate key formulation parameters, i.e., dissolution behavior. The novelty of this work lies in developing an integrated computational framework that directly links miscibility parameters and MD-simulated CED values to printability outcomes and dissolution behavior, respectively. A central focus is the identification of suitable excipients, optimal drug-to-excipient ratios, and material properties that collectively influence both miscibility and printability, thereby enabling informed selection of formulation components. The overarching goal is to demonstrate the utility of in silico approaches for reducing the risks during early-stage formulation development and to establish a framework for integrating data-driven predictive insights into the design and optimization of 3DP-DDS.

## 2. Materials and Methods

The excipient structures were drawn and optimized using ChemDraw^®^ Ultra version 10 (Cambridgesoft, Waltham, MA, USA). The in silico study was performed using Materials Studio modeling suite (v2017, Accelrys Inc., San Diego, CA, USA). Correlation analyses and graphs were generated using GraphPad Prism (version 9, GraphPad Software, Inc., Boston, MA, USA).

### 2.1. 3D-DDS Data Mining and Collection

Formulation composition data were compiled into a structured dataset (Table 1 and Table 2). For each formulation, we recorded the API identity and load, the excipients (polymer(s), plasticizer(s), surfactant(s), and filler(s)) along with their exact weight/weight percentages, and the hot-melt extrusion (HME) processing temperature. For printability prediction, the final extrusion temperature was considered. Only studies reporting complete composition data in which the component percentages summed to 100% *w*/*w* were included, whereas records with missing, ambiguous, or inconsistent data were excluded. Formulation compositions and printability outcomes were obtained from previously published studies, and all corresponding citations are provided in the footnotes of Table 1 and Table 2.

### 2.2. Predictability in Two Stages of 3DP-DDS

The in silico framework focuses on two critical stages in the development of 3D-printed drug delivery systems (3DP-DDS):

#### 2.2.1. Stage 1. Printability

The model predicts the “non-printable/printable window” by employing E_mix_ and χ (chi) values using the blend module for the selected excipient–API system. In addition, it assesses filament quality, specifically evaluating whether the extrudate is likely to be soft or brittle.

#### 2.2.2. Stage 2. Dissolution Profile

All printed 3D-DDS compositions (API–excipient mixture systems) were subjected to MD simulations to calculate the CED values. These values were used to evaluate the dissolution behavior of the formulations and were correlated with the reported T80% (time to 80% drug release).

### 2.3. API and Excipient Systems Preparation

#### 2.3.1. Preparation of the Chemical Structures of API and Excipients

API 3D structures were sourced from the ChemSpider platform [60], while the excipient structures were drawn and optimized using ChemDraw^®^ Ultra version 10 (Cambridgesoft, Waltham, MA, USA). To maintain computational feasibility, excipients were represented by a reduced number of repeating units (*n*) compared to their actual polymer lengths. The *n* values were adjusted according to the relative molecular weight of low-, medium-, and high-grade polymers. The exact *n* values assigned to each excipient are provided in the corresponding Table 1 and Table 2 footnotes.

#### 2.3.2. Structure Preparation

Molecular structures of the API and excipients were prepared using the Materials Studio modeling suite (v2017, Accelrys Inc., San Diego, CA, USA). Energy minimization of the API and excipients was initially carried out, and their geometries were subsequently optimized using the Forcite module with the Dreiding force field.

#### 2.3.3. Excipient and API System Construction Using Amorphous Cell Module

For the miscibility study, excipient and API systems were prepared at their respective reported formulation ratios using the Amorphous Cell module in the Materials Studio modeling suite (v2017, Accelrys Inc., San Diego, CA, USA). The API system was constructed under periodic boundary conditions (PBC) at the reported ratios and HME temperature. The excipient system, comprising all excipients, was built separately in an independent PBC box at the reported ratio and HME temperature. The amorphous cells were generated using a cubic unit cell configuration, with the dimensions represented by the vectors a, b, and c (Å) (Table 1). An initial cell was created at a predefined density (g/cm^3^) through the ramp-density function. This starting structure was then progressively adjusted toward the target density defined in the setup panel, using repeated rounds of geometry optimization to stabilize the system.

For the MD simulations, a similar procedure as described for the miscibility study was followed, wherein the API–excipient mixture was generated at the reported ratio and HME temperature using the Amorphous Cell module, ensuring consistency in construction parameters across all systems (Table 2) [61,62].

### 2.4. Printability Prediction

#### Miscibility Parameters for Predicting Printability

Energy minimization of the API and excipient systems was first performed, followed by geometry optimization using the Forcite module with the Dreiding force field. The Blend module was then employed to calculate the mixing energy (E_mix_) and FH interaction parameter (χ) at the HME processing temperature, using medium-quality settings and current charges. These parameters were used to assess the miscibility of the API–excipient combinations at the given ratios.

The mixing energy, representing the difference between the mixture and the sum of the pure component energies, was estimated as Equation (1):

(1) $$E_{mix}=\frac{1}{2}Z(E_{bs}+E_{sb}-E_{bb}-E_{ss})$$

The interaction parameter χ, describing drug–excipient affinity, was derived as Equation (2):

(2) $$\chi =\frac{E_{mix}}{R.T}$$

where R is the universal gas constant and T is the absolute temperature. Negative E_mix_ values indicate favorable miscibility. The lower value chi (χ) of the higher mutual interaction (i.e., miscibility) of the API–excipients system [63,64,65]. Table 1 summarizes the predicted miscibility parameters (E_mix_ and χ) of different API and excipient systems assessed through the blend module for printability prediction at the reported HME temperature.

### 2.5. Dissolution Prediction

#### Cohesive Energy Density for Predicting Dissolution Behavior

API–excipient mixture systems were prepared using the Amorphous Cell tool, as summarized in Table 2. MD simulations were carried out following several steps using the Materials Studio modeling suite (version 2017, Accelrys Inc., San Diego, CA, USA). First, energy minimization was performed for all systems, employing the Dreiding force field to model atomic interactions. The computed densities showed close consistency with previously reported experimental values (g/cm^3^). A time step of 1 fs was employed for integration. Long-range interactions were evaluated using the Ewald summation method, applying a 6.0 Å cutoff for both electrostatic and non-bonded forces. Simulations were initially performed in the NPT ensemble (constant number of particles, pressure, and temperature) at the reported printing temperatures for 100 ps to obtain equilibrium densities. The production run was then conducted in the NVT ensemble (constant number of particles, volume, and temperature) for 500 ps. Equilibration was assessed by monitoring thermodynamic properties (energies, temperatures, and densities) over time. A system was considered equilibrated when these properties exhibited minimal fluctuations, which occurred in less than 200 ps for all systems. Finally, the CED was calculated from five independent trajectory datasets obtained from the equilibrated systems [66,67,68].

## 3. Results and Discussion

Experimental techniques and analytical methodologies commonly used to predict the physicochemical behavior of 3D-printed drug products, such as printability assessments, filament extrusion trials, thermal analysis, mechanical testing, and dissolution studies, are often time-consuming and resource-intensive [14,69]. As a result, relying solely on conventional wet-lab approaches for early-stage formulation screening can significantly delay development timelines, increase material consumption, and require repeated optimization cycles before a viable 3D-printed formulation is identified [70,71].

In silico predictions during formulation development provide an effective means to reduce experimental workload and enable the rational selection of excipients and drug-to-excipient ratios, provided with appropriate computational tools. In this study, we utilized the Materials Studio Blend module and MD simulations, which are well-established techniques for investigating the molecular interaction of two or more components. For the first time, the Blend module was employed to predict the printability of 3DP-DDS, while MD simulations were used to compute CED to forecast the dissolution behavior of the printed formulations. Our work goes beyond conventional miscibility analysis by introducing a comprehensive strategy that integrates in silico miscibility data with MD simulation outputs to predict the performance of 3DP-DDS from initial formulation development through to final dissolution behavior.

### 3.1. Printability Prediction

Drug–excipient miscibility is the primary determinant of whether a formulation is printable, while printer settings such as nozzle diameter, layer height, speed, and pressure act as secondary tuning knobs that refine shape fidelity once a printable ink has been achieved. Poor miscibility often leads to drug crystallization, irregular melt viscosity, nozzle clogging, and poor mechanical integrity of printed structures [15,72]. In contrast, once a stable and miscible system is achieved, process settings such as nozzle diameter and layer height primarily influence how the material is deposited, governing strand continuity, resolution, porosity, and overall geometric fidelity of the printed construct [73,74].

**Figure 1:**
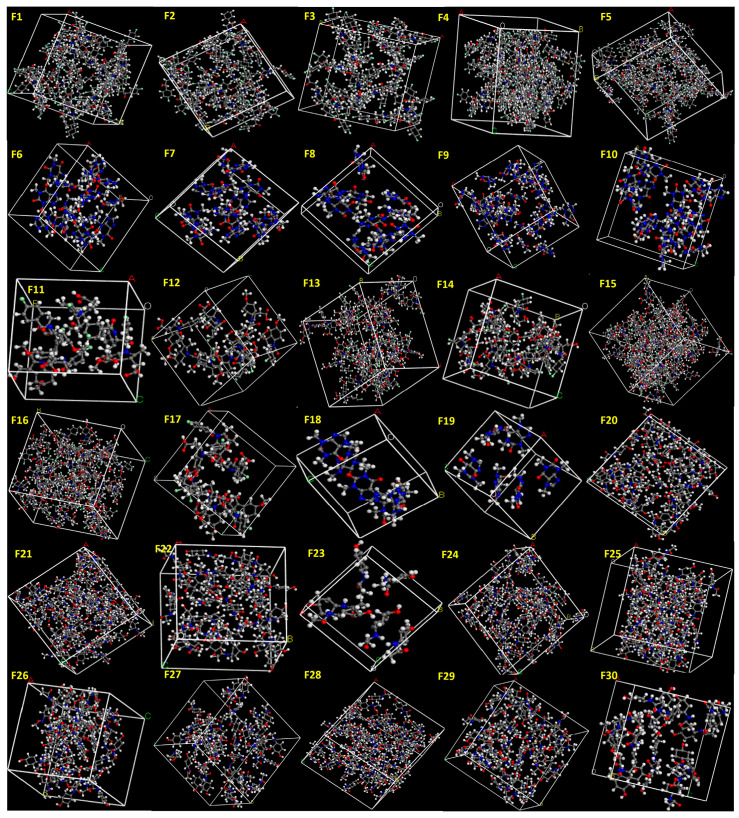
API systems (Formulations F1–F30) constructed at the reported ratios using the Amorphous Cell module. The generated structures were employed to determine E_mix_ and χ values for assessing formulation printability.

**Figure 2:**
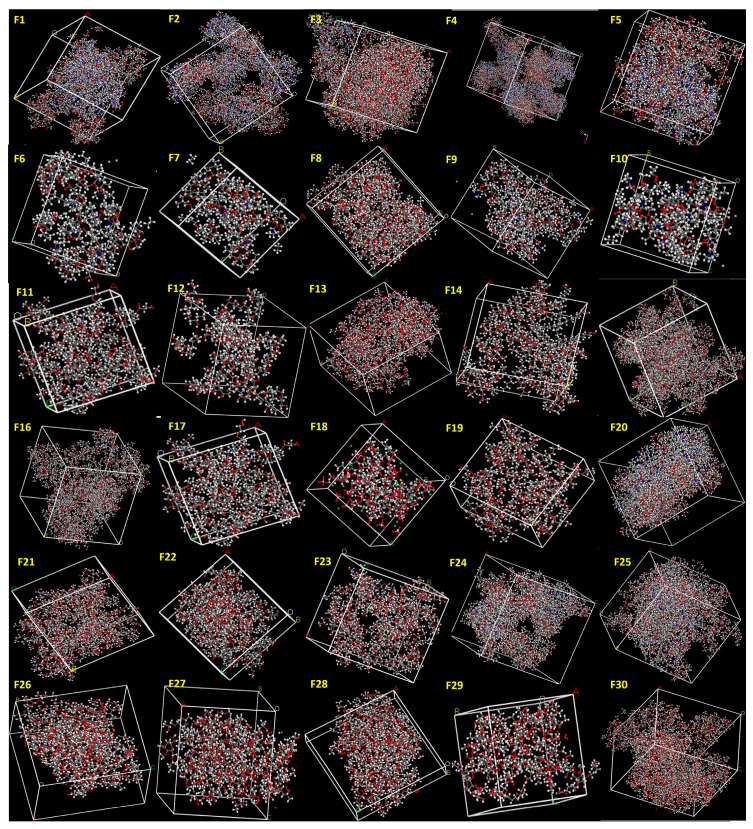
Excipient systems (Formulations F1–F30) constructed at the reported ratios using the Amorphous Cell module. The generated structures were employed to determine E_mix_ and χ (chi) values for assessing formulation printability.

In this study, API and excipient systems were constructed in amorphous cells at the reported concentrations, respectively (Figure 1 and Figure 2). The Amorphous Cell approach was selected because it enables the generation of realistic, disordered molecular structures that closely resemble the amorphous state produced during HME. Previous studies commonly evaluated miscibility using only one drug molecule and one excipient molecule, which does not reflect true formulation compositions and often fails to predict experimental outcomes. In contrast, our approach incorporates accurate incorporation of the API and excipients at their exact experimental ratios, ensuring that the simulated systems directly reflect the actual formulation compositions (Figure 1 and Figure 2), thereby providing a more realistic molecular model that aligns closely with experimental printability results.

The Dreiding forcefield was used as it provides broad element coverage, transferable parameterization, and consistent description of intermolecular interactions, which is important for accurate miscibility calculation [75]. Initial trials with COMPASS and other forcefields did not produce reproducible results, mainly due to missing atom-type parameters for the API–excipient and tendency to cause convergence problem or yield unrealistic interaction energies in mixed amorphous systems. In contrast, Dreiding provided stable energy minimization, reliable non-bonded interactions, and consistent thermodynamic outputs across all compositions [76,77]. The Blend module was specifically used to determine the FH interaction parameter (χ) and mixing energy (E_mix_), which serve as robust indicators of the molecular compatibility between the API and excipients [78]. All calculations were performed at the corresponding HME processing temperatures to closely replicate the actual conditions of formulation and 3D printing.

Importantly, when the same calculations were performed at 25 °C (ambient temperature), several formulations were falsely predicted as miscible. These systems did not correlate with experimental results and were not printable in practice. This finding highlights the critical importance of consideration of the actual processing temperature in in silico miscibility parameters. Drug–excipient molecular interactions, solubility, and molecular mobility change significantly with temperature, particularly in HME and 3D printing processes, where elevated temperatures can enhance extrudability and printability [79,80,81]. Therefore, predictions made at ambient conditions may not accurately reflect behavior during processing.

To assess the predictive reliability of this approach, we examined a variety of 3DP formulations, including acid–base combinations, plasticizer-containing formulations, different grades of the same polymer, and systems with small variations in drug–excipient ratios (Table 3). In all these cases, changes that influenced printability were accurately predicted by the in silico miscibility study. For instance, formulation involving acid–base interaction-type exhibited predicted miscibility that aligned with experimentally observed outcomes. Incorporating an acidic component into the drug–excipient system led to negative E_mix_ and χ values, signifying high miscibility and correlating with successful printing results (Table 3).

Furthermore, the presence of plasticizers (such as stearic acid and PEG) improved miscibility, as evidenced by highly negative χ and E_mix_ values (Table 3). These excipients likely promote favorable hydrophobic and hydrophilic interactions, enhancing overall compatibility between drug and polymer, thereby facilitating successful extrusion and printing. Formulations employing the same polymer but different plasticizers exhibited distinct miscibility profiles, underscoring the critical influence of excipient selection on formulation performance. Even small variations in excipient concentration were found to impact printability, which correlated precisely with significant increase in the negative χ and E_mix_ values (Table 3).

**Figure 3:**
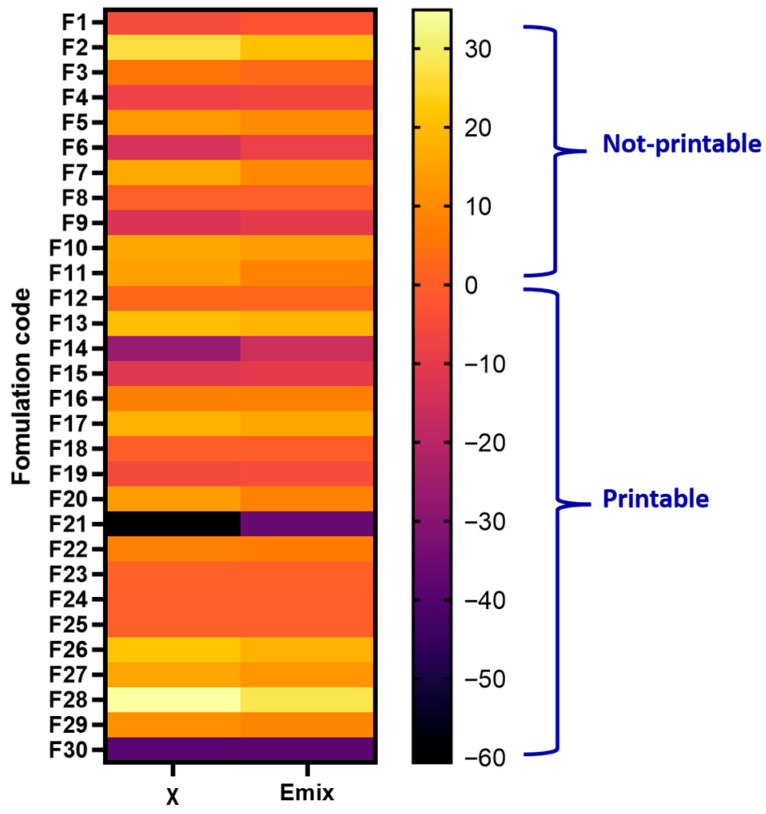
Heatmap of FH interaction parameter (χ) and mixing energy (E_mix_) for 30 formulations (API–excipients system) (F1–F30). Cooler colors (purple to black) indicate strongly negative values, corresponding to higher predicted miscibility, while warmer colors (orange to yellow) represent positive values, indicating poor miscibility.

The results revealed that formulations with negative χ (<−5) and negative E_mix_ (<−5) values were consistently predicted to be highly miscible, and these predictions aligned with successful printability outcomes. In contrast, systems with positive χ (>−1) and positive E_mix_ (>−1) values were predicted to be immiscible and were reported as non-printable, indicating poor drug–excipient compatibility (Figure 3). Receiver Operating Characteristic (ROC) evaluation (AUC = 1.00) revealed that χ and E_mix_ < −5 maximizes Youden’s J statistic and provides perfect separation of printable and non-printable samples within this dataset (100% sensitivity and 100% specificity). This result confirms that highly negative χ and E_mix_ values are strongly associated with experimentally printable formulations and supports using E_mix_ and χ ≈ −5 as a practical miscibility boundary.

**Figure 4:**
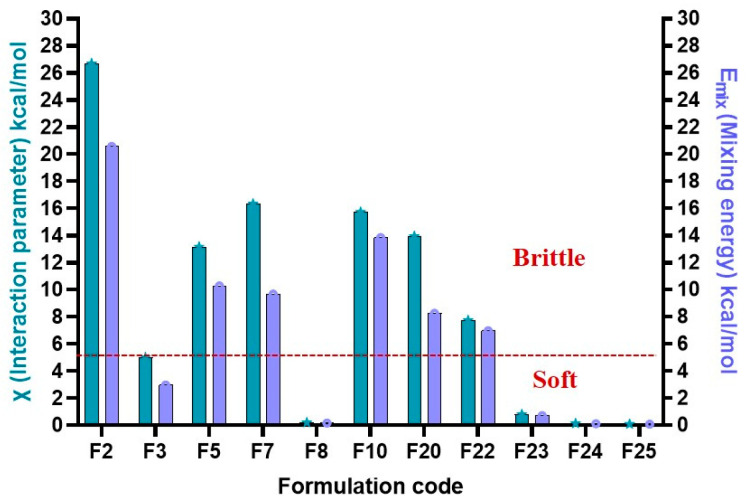
Prediction window for 3DP-DDS physical quality (brittle vs. Soft) based on E_mix_ (mixing energy) and χ (FH interaction parameter) values.

A predictive window for formulation behavior during 3D printing was identified using E_mix_ and χ values. Mildly negative to near-zero E_mix_ values (−5 to 0) produced soft but non-printable formulations, consistent with excessive miscibility and plasticization that lowered modulus and prevented structural retention. Positive E_mix_ values (>+5) yielded brittle filaments due to poor miscibility and phase separation, while strongly negative values (≤−5) consistently generated printable filaments, reflecting a balance between chain mobility and rigidity, as illustrated in Figure 4. These trends align with FH theory, where χ describes the relative contribution of enthalpic and entropic interactions to miscibility. Negative χ values favor miscibility through adhesive interactions, whereas positive χ values indicate dominant cohesive interactions leading to phase separation [82].

**Figure 5:**
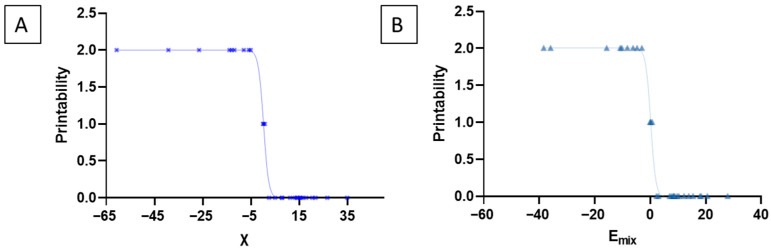
These ordinal printability scores were correlated with the predicted miscibility parameters to evaluate the relationship between miscibility parameters and experimental printability. Printability was categorized as 2 = printable (highly miscible, mechanically stable filaments), 1 = soft or partially printable (low structural integrity), and 0 = brittle/non-printable. Blue cross represents the formulation F1 to F30. Light green triangles represent the formulation F1 to F30. (**A**) Plot of χ versus printability. (**B**) Plot of E_mix_ versus printability.

To evaluate the strength of association between miscibility parameters and experimental printability, non-linear regression and binary classification analyses were performed to establish the presence of strong correlations. Consistent with this behavior, non-linear regression revealed a strong correlation between E_mix_ and printability (R^2^ = 0.994, RMSE = 0.069), and binary classification after grouping printability scores 0–1 as non-printable and 2 as printable achieved an accuracy of 86.7% with perfect sensitivity (100%) and high specificity (81%). Similarly, χ-based non-linear regression also demonstrated a strong predictive relationship (R^2^ = 0.99, RMSE = 0.066), yielding 90% accuracy with perfect sensitivity (100%) and high specificity (85.7%). As illustrated in Figure 5, these ordinal printability scores (2 = printable, 1 = soft/partially printable, and 0 = brittle/non-printable) were correlated with χ and E_mix_ values to establish the relationship between predicted miscibility parameters and experimental printability.

Overall, the results confirmed that in silico miscibility screening, when performed at processing temperatures, is a reliable tool for predicting the success of 3DP pharmaceutical formulations. It can help to reduce experimental workload by identifying promising API–excipient combinations early in the development process.

### 3.2. Dissolution Behavior Prediction

In 3DP-DDS, dissolution behavior is largely governed by API–excipients interaction, filament properties, and formulation design features such as geometry, porosity, and infill density [70,83]. Nevertheless, in 3DP-DDS, composition plays a more decisive role than geometry in determining dissolution behavior, as it governs the fundamental drug release mechanism through factors such as polymer type, drug solubility, and excipient interactions [84]. The choice of matrix materials (e.g., hydrophilic vs. hydrophobic polymers) strongly influences whether release occurs via diffusion, erosion, or swelling [85,86]. Geometry through variables such as surface area, infill density, and shape modulates the rate of dissolution by affecting fluid penetration and exposure, but it cannot override the release pathway dictated by the formulation [87,88]. Thus, composition establishes the foundation of drug release, while geometry serves as a secondary tool for fine-tuning the dissolution profile.

Drug release and dissolution testing remain challenging across many pharmaceutical formulations because release behavior is highly sensitive to formulation composition, microstructure, processing conditions, and testing variability [89]. Conventional dissolution experiments are often time-consuming, labor-intensive, and low-throughput, especially when multiple prototypes or process parameters must be screened [90]. In this context, in silico and computational prediction tools, e.g., MD simulation, modeling drug release kinetics, machine learning, mechanistic diffusion–erosion models, and PBPK simulations, have become increasingly valuable. These approaches can predict dissolution trends, identify critical formulation parameters, reduce experimental workload, and accelerate development by narrowing down the most promising formulations before laboratory testing [91,92].

In silico predictions based on CED provide valuable insights into molecular interactions that directly influence drug release profiles [93]. Such a computational tool is particularly important for 3DP systems, where dissolution is highly variable and often non-linear. By contrast, conventional dosage forms exhibit relatively uniform microstructures and predictable release mechanisms, where dissolution can be effectively assessed through routine in-vitro testing without the need for extensive computational modeling [94,95]. CED represents the energy required to separate molecules from each other within a formulation. A high CED indicates strong drug–polymer interactions (e.g., hydrogen bonding, van der Waals, and hydrophobic contacts). Across all systems investigated, a consistent relationship was observed: higher CED values correlated with stronger binding and delayed release, while lower values reflected weaker interactions and faster dissolution. Together, CED acts as a molecular fingerprint that can predict whether a formulation will behave as an immediate-release or sustained-release system, even before experimental validation.

#### 3.2.1. Dissolution Prediction: General Trends Across Polymers

**Figure 6:**
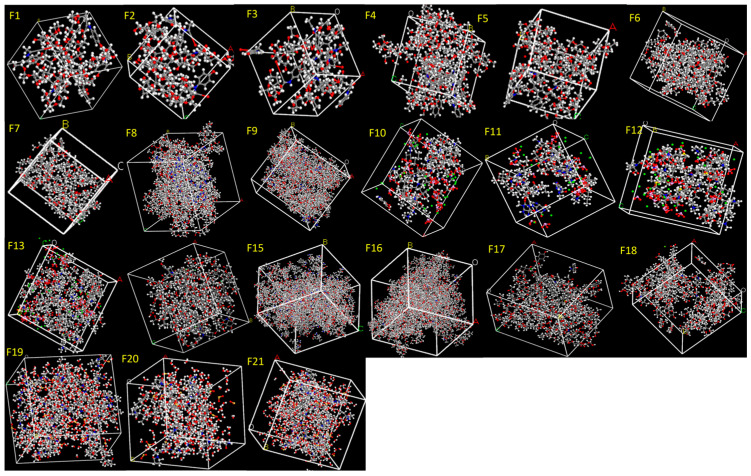
API–excipient systems (Formulations F1–F21) constructed at the reported ratios using the Amorphous Cell module. The generated structures were subjected to MD simulations to calculate the cohesive energy density (CED).

In this study, API–excipient systems were constructed in amorphous cells at the reported concentrations and corresponding HME processing temperatures to closely replicate the actual conditions of formulations and 3D printing (Figure 6). The Dreiding force field was selected because it provided stable outputs across all compositions. MD simulations were subsequently performed at 310 K to represent the dissolution conditions.

Hydrophilic polymers, such as Kollicoat IR and HPMC, exhibited lower CED corresponding to faster drug release. In contrast, more hydrophobic carriers, such as polycaprolactone (PCL), or functional excipients like HPMCAS-HG, revealed high CED values, indicating stronger drug entrapment and sustained release. Multi-polymer blends (e.g., Kollidon^®^ VA 64/PCL systems) generated exceptionally high interaction values, predicting very slow release, while drug-specific variation was also evident within the same polymer base (e.g., Eudragit EPO formulations), as summarized in Table 4.

Drug concentration strongly influenced dissolution. In Kollicoat IR–paracetamol systems, increasing drug loading from 5% to 15% progressively raised CED (16.3–120 × 10^8^ J/cm^3^), leading to slower release. These results demonstrate that drug loading can either strengthen or weaken interactions depending on drug–polymer compatibility [67]. Hydrophobic matrices such as PCL systems showed very high interaction strength (CED 94.4–172 × 10^8^ J/cm^3^), confirming their role in sustained release [53]. In contrast, hydrophilic polymers like HPMC allowed faster hydration and diffusion, though increasing polymer proportion (e.g., from 30% to 50% HPMC) enhanced CED (33.6–50 × 10^8^ J/cm^3^), extending release times [52]. Thus, the balance of hydrophilicity and hydrophobicity is a key determinant of dissolution profiles.

Eudragit EPO systems highlighted API-dependent effects: theophylline produced higher CED (79.4 × 10^8^ J/cm^3^) compared to prednisolone or captopril (CED ~57–59 × 10^8^ J/cm^3^), showing that drug chemistry significantly modulates release within the same matrix [55]. Furthermore, formulations combining Kollidon^®^ VA 64 + PCL systems generated extremely high CED values (166–259 × 10^8^ J/cm^3^), predicting markedly delayed dissolution [54]. This illustrates how polymer blending can be used to fine-tune matrix hydrophobicity and achieve desired release profiles beyond what is possible with a single polymer.

**Figure 7:**
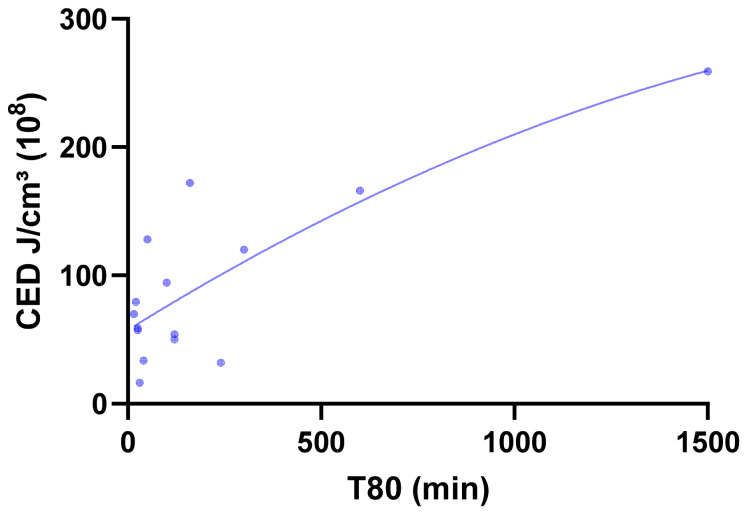
Non-linear (second-order polynomial) regression curve describing the relationship between CED (J/cm^3^ ×10^8^) and T_80_ dissolution time (min) for formulations F1–F15.

The relationships were quantitatively confirmed through a non-linear (second-order polynomial) regression analysis between the calculated CED and the experimental dissolution parameter T_80_ (Figure 7). Across all 15 formulations, a strong non-linear correlation was obtained (R^2^ = 0.8958, RMSE = 120.31 min), demonstrating that CED is a reliable predictor of release behavior across diverse polymer systems. Binary classification uses a median T_80_ (≈100 min) threshold for slow (T_80_ > median) vs. fast release, with Random Forest achieving the highest accuracy (86.7%) and specificity (87.5%).

#### 3.2.2. Polymer Grade-Dependent Trends in Dissolution Prediction

**Figure 8:**
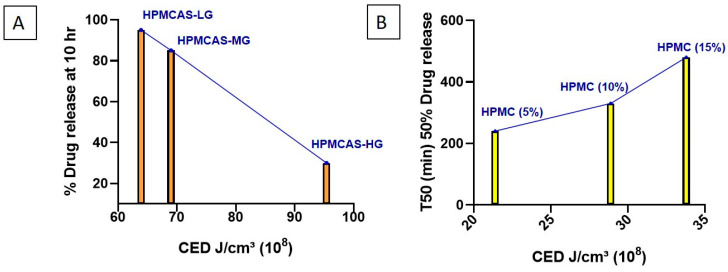
MD-simulated cohesive energy density (CED) values and comparisons for (**A**) HPMCAS grades LG, MG, and HG, and (**B**) HPMC at 5%, 10%, and 15% *w*/*w*.

The effect of HPMCAS grade (LG, MG, HG) on the dissolution behavior of formulations was evaluated. The % of drug released at 10 h and the CED for each formulation are summarized in Table 4. HPMCAS-HG exhibited the highest CED, indicative of a greater degree of hydrophobicity and stronger drug–polymer interactions. This resulted in markedly slower dissolution, with only 30% of the drug released at 10 h. In contrast, HPMCAS-LG demonstrated the lowest CED (63.9 × 10^8^), corresponding to higher hydrophilicity and rapid drug release (95% at 10 h). HPMCAS-MG displayed intermediate CED (69 × 10^8^), resulting in a moderate release of 85% at 10 h (Figure 8A). The observed trend correlates with the known substitution patterns of the grades. HG contains relatively higher acetyl (7–8%) and succinoyl (~13–15%) content, which increases hydrophobicity and raises the pH threshold for dissolution. MG (acetyl 8–9%, succinoyl 11–13%) and LG (acetyl 9–10%, succinoyl 10–12%) are comparatively more hydrophilic, leading to earlier hydration and faster dissolution [96,97,98]. These findings confirm the relationship between polymer hydrophobicity and drug release: increased hydrophobicity enhances drug–polymer interactions and delays dissolution, whereas greater hydrophilicity promotes rapid drug release. This relationship enables rational selection of HPMCAS grade to achieve the desired site-specific release in the gastrointestinal tract.

#### 3.2.3. Polymer Concentration-Dependent Trends in Dissolution Prediction

To further examine the predictive utility of CED values, three formulations containing varying concentrations of the same HPMC grade (5%, 10%, and 15%) were analyzed (Table 4). The formulation with 5% HPMC exhibited the lowest CED (21.4 × 10^8^ J/cm^3^), which correlated with faster dissolution (50% drug release within 240 min). In contrast, increasing the HPMC concentration to 10% and 15% progressively elevated CED 28.9 and 33.8 × 10^8^ J/cm^3^, respectively, resulting in slower dissolution (50% release at 330 and 480 min, respectively) (Figure 8B).

These findings suggest that increasing HPMC content, while reducing Kollidon SR proportion, strengthens drug–polymer interactions and thereby delays dissolution. Taken together with the results for different polymer grades and concentrations, this study demonstrates that CED values provide a reliable predictive approach for anticipating the dissolution behavior of polymer-based formulations.

The data validates the feasibility of an in silico approach for predicting excipients that can enable the printability of 3D-DDS. A noteworthy outcome of the study is the correlation between in silico prediction and actual experimental results. Positive E_mix_ and χ values in the miscibility study are predictors of the non-printability and serve as a basis to reject the excipient, and negative values indicate predominance of the printability, providing options for selection. The MD simulation CED value could add value by enabling an understanding of the dissolution behaviors of 3DP-DDS, to assist in drug-to-exipient ratio, polymer grade selection, and excipient type.

The miscibility parameters and CED-based models used in this work are fundamentally developed for binary systems, such as drug–polymer or drug–drug mixtures. Therefore, their transferability to multi-drug formulations or systems containing multiple excipients is inherently restricted [99]. In multi-drug formulations, multiple interactions, such as drug–drug, drug–polymer, and drug–excipient, take place concurrently, making it difficult for simple pairwise parameters to describe competitive hydrogen bonding, phase separation, or crystallization risks [100]. As a result, predicting printability becomes challenging for complex, multi-component 3D-printed systems.

Moreover, the applicability of miscibility parameters is largely limited to FDM-based 3D printing, where melt behavior and thermal drug–polymer miscibility govern printability. When alternative techniques, such as SLA or SSE, are used, the underlying mechanisms differ substantially. SLA technique relies on photopolymerization rather than melt blending, and SSE depends on rheology and pressure-driven flow [74].

## 4. Conclusions

This study investigated the correlation of miscibility and CED with the printability and dissolution behavior, respectively, demonstrating the utility of an in silico-driven approach for 3D-printed formulations. The application of this in silico framework enables a rapid and reliable predictive screening model for designing 3D-DDS. Bridging gaps in the conventional formulation optimization process, this emerges as a major driver of computer-aided drug development, with strong potential to enhance efficiency, streamline excipient screening, and accelerate next-generation pharmaceutical innovation.

## Figures and Tables

**Table 1:** Miscibility prediction of different API and excipient systems using the Materials Studio blend module (E_mix_ and χ) for assessing printability. (D—API system, and E—excipient system).

Formulation Code	API System (D)	Excipient System (E)	Temp (°C)	Amorphous Cell Lengths (a (Å), b (Å), c (Å)
F1	Haloperidol (15%)	Kollidon^®^ VA 64 (BASF SE, Ludwigshafen, Germany) (74.5%) + glutaric acid (10.5%)	115	D-29.2 × 29.2 × 29.2 E-50.4 × 50.4 × 50.4
F2	Haloperidol (15%)	Kollidon^®^ VA 64 (85%)	150	D-29.2 × 29.2 × 29.2 E-52.0 × 52.0 × 52.0
F3	Haloperidol (15%)	Kollidon^®^ VA 64 and Affinisol™ 15Cp (The Dow Chemical Company, Midland, MI, USA) (4:6) (74.5%) + glutaric acid (10.5%)	115	D-28.2 × 28.2 × 28.2 E-50.0 × 50.0 × 50.0
F4	Haloperidol (30%)	Kollidon^®^ VA 64 (79.6%) + malic acid (5.4%)	120	D-33.5 × 33.5 × 33.5 E-59.4 × 59.4 × 59.4
F5	Haloperidol (40%)	Kollidon^®^ VA 64 (31.5%) + malic acid (28.5%)	120	D-35.5 × 35.5 × 35.5 E-34.3 × 34.3 × 34.3
F6	Theophylline (30%)	Eudragit RLPO (Evonik Industries AG, Darmstadt, Germany) (62.6%) + stearic acid (7%)	180	D-20.0 × 20.0 × 20.0 E-27.0 × 27.0 × 27.0
F7	Theophylline (30%)	Eudragit RLPO (70%)	175	D-19.6 × 19.6 × 19.6 E-25.8 × 25.8 × 25.8
F8	Theophylline (9.45%)	HPC MF (84.69%) + PEG300 (5.48%)	145	D-17.6 × 17.6 × 17.6 E-37.7 × 37.7 × 37.7
F9	Theophylline (30%)	Eudragit RLPO (59.6%) + PEG4000 (10%)	145	D-23.2 × 23.2 × 23.2 E-29.8 × 29.8 × 29.8
F10	Theophylline (30%)	Eudragit RLPO (66.1%) + Stearic acid (3.5%)	170	D-20.0 × 20.0 × 20.0 E-26.1 × 26.1 × 26.1
F11	Indomethacin (30%)	HPMC-AS HG (65%) + HPC LF (5%)	160	D-18.1 × 18.1 × 18.1 E-28.8 × 28.8 × 28.8
F12	Indomethacin (30%)	PVA (70%)	160	D-20.7 × 20.7 × 20.7 E-27.5 × 27.5 × 27.5
F13	Indomethacin (30%)	HPMC K15M (70%)	180	D-32.9 × 32.9 × 32.9 E-43.7 × 43.7 × 43.7
F14	Indomethacin (30%)	HPMC K15M (60%) + Stearic acid (10%)	180	D-18.1 × 18.1 × 18.1 E-35.4 × 35.4 × 35.4
F15	Indomethacin (30%)	HPMCAS-HG (60%) + PEG 4000 (10%)	160	D-35.1 × 35.1 × 35.1 E-46.3 × 46.3 × 46.3
F16	Indomethacin (30%)	HPMCAS-HG (70%)	160	D-35.2 × 35.2 × 35.2 E-49.5 × 49.5 × 49.5
F17	Indomethacin (30%)	HPMCAS-HG (65%) + HPC-LF (5%)	160	D-10.2 × 10.2 × 10.2 E-30.3 × 30.3 × 30.3
F18	Caffeine (10.03%)	HPC-SSL (37.34%) + PEG4000 (15.24%) + Dicalcium phosphate (37.34%)	120–145	D-13.9 × 13.9 × 13.9 E-26.1 × 26.1 × 26.1
F19	Caffeine (10.03%)	HPC-SSL (89.97%)	120–145	D:14.8 × 14.8 × 14.8 E-22.6 × 22.6 × 22.6
F20	Acetaminophen (30%)	HPMC E5 (45.5%) + Soluplus (15%)	180	E-50.2 × 50.2 × 50.2 D-32.4 × 32.4 × 32.4
F21	Acetaminophen (30%)	HPMC E5 (45.5%) + HPC LF (15%)	180	D:13.6 × 13.6 × 13.6 E-33.4 × 33.4 × 33.4
F22	Acetaminophen (30%)	HPMC E5 (35%) + Eudragit L100 (35%)	180	D-26.2 × 26.2 × 26.2 E-38.6 × 38.6 × 38.6
F23	Acetaminophen (30%)	HPMC E5 (35%) + HPC LF (35%)	180	D-38.8 × 38.8 × 38.8 E-41.1 × 41.1 × 41.1
F24	Acetaminophen (30%)	HPC LF (35%) + Ethyl cellulose (35%)	160	D-29.6 × 29.6 × 29.6 E-38.7 × 38.7 × 38.7
F25	Acetaminophen (30%)	Ethyl cellulose (35%) + Soluplus (35%)	160	E-49.9 × 49.9 × 49.9 D-30.5 × 30.5 × 30.5
F26	Paracetamol (25%)	Affinisol™ (The Dow Chemical Company, Midland, MI, USA) 15LV (75%)	130	D-28.3 × 28.3 × 28.3 E-38.2 × 38.2 × 38.2
F27	Paracetamol (30%)	Affinisol™ 15LV (70%)	130	D-26.2 × 26.2 × 26.2 E-34.7 × 34.7 × 34.7
F28	Paracetamol (35%)	Affinisol™ 15LV (65%)	110	D-31.1 × 31.3 × 31.1 E-38.2 × 38.2 × 38.2
F29	Paracetamol (40%)	Affinisol™ 15LV (60%)	110	D-26.4 × 26.4 × 26.4 E-30.3 × 30.3 × 30.3
F30	Paracetamol (5%)	Affinisol™ 15LV (95%)	180	D-19.6 × 19.6 × 19.6 E-55.1 × 55.1 × 55.1

Formulation compositions were adapted from the published literature (see corresponding references): F1–F3 [44], F4–F5 [45], F6–F10 [46], F11–F17 [47], F18–F19 [48], F20–F25 [49], and F26–F30 [50]. *n* = number of repeating units modeled for each excipient oligomer. For computational feasibility, large polymers were represented by shorter oligomer chains. Representative oligomers were constructed as follows: Eudragit RLPO, Kollidon^®^ VA64 (vinylpyrrolidone (6)/vinyl acetate (4)): constructed according to given molecular weight; HPC grades: HPC-SSL (*n* = 2), HPC-LF (*n* = 4), and HPC-MF (*n* = 8); HPMC grades: HPMC E5 (*n* = 4), HPMC 15 (*n* = 8), and HPMC K15M (*n* = 10), Affinisol™ 15LV (*n* = 10); Ethyl cellulose, HPMCAS (HG/MG/LG): constructed according to given molecular weights; Soluplus (PEG 6000 (13)/vinylcaprolactam (57)/vinyl acetate (3)): constructed according to given molecular weights; PEG: PEG4000 (*n* = 400), and PEG300 (*n* = 30).

**Table 2:** CED estimation using MD simulation of API–excipients mixture systems using Materials Studio Forcite module for predicting dissolution.

Formulation Code	Polymer Matrix	Formulation Composition	Amorphous Cell Lengths (a (Å), b (Å), c (Å))	Temp (°C)
F1	Kollicoat IR	Paracetamol (PCM) (5%) + Kollicoat IR (BASF SE, Ludwigshafen, Germany) (92%)	17.2 × 17.2 × 17.2	110
F2		PCM (10%) + Kollicoat IR (77%)	17.2 × 17.2 × 17.2	110
F3		PCM (15%) + Kollicoat IR (62%)	17.2 × 17.2 × 17.2	110
F4	HPMC K4M	Naftopidil (20%) + HPMC (30%) + Mannitol (45%) + PEG4000 (10%)	18.7 × 18.7 × 18.7	90
F5		Naftopidil (20%) + HPMC (50%) + Mannitol (15%) + PEG4000 (10%)	20.0 × 20.0 × 20.0	90
F6	Polycaprolactone (PCL)	Acetylsalicylic acid (10%) + PCL (90%)	29.3 × 29.3 × 29.3	100
F7		Acetylsalicylic acid (15%) + PCL (85%)	27.0 × 27.0 × 27.0	100
F8	Kollidon^®^ VA 64	Caffeine (5%) + Kollidon^®^ VA 64 (40%) + PCL (45%) + PEO (25%)	48.1 × 48.1 × 48.1	140
F9		Caffeine (5%) + Kollidon^®^ VA 64 (30%) + PCL (55%) + PEO (10%)	44.0 × 44.0 × 44.0	140
F10	Eudragit EPO	5-aminosalicylic acid (12.5%) + Eudragit EPO (Evonik Industries AG, Darmstadt, Germany) (46.75%) + Triethyl citrate (3.25%) + Tricalcium phosphate (37.5%)	27.5 × 27.5 × 27.5	90–100
F11		Theophylline (12.5%) + Eudragit EPO (46.75%) + Triethyl citrate (3.25%) + Tricalcium phosphate (37.5%)	26.3 × 26.3 × 26.3	90–100
F12		Captopril (12.5%) + Eudragit EPO (46.75%) + Triethyl citrate (3.25%) + Tricalcium phosphate (37.5%)	29.1 × 29.1 × 29.1	90–100
F13		Prednisolone (12.5%) + Eudragit EPO (46.75%) + Triethyl citrate (3.25%) + Tricalcium phosphate (37.5%)	34.6 × 34.6 × 34.6	90–100
F14	HPMCAS-HG	Indomethacin (20%) + HPMCAS-HG (60%) + PEG6000 (20%)	30.8 × 30.8 × 30.8	140
F15		Pregabalin (50%) + HPMCAS-HG (40%) + PEG 400 (10%)	34.9 × 34.9 × 34.9	140
Polymer Grades				
F16	HPMCAS-HG	PCM (5%) + HPMCAS-HG (95%) + Methyl paraben (15%) + Magnesium stearate (5%)	50.4 × 50.4 × 50.4	-
F17	HPMCAS-MG	PCM (5%) + HPMCAS-MG (95%) + Methyl paraben (15%) + Magnesium stearate (5%)-MG	33.9 × 33.9 × 33.9	-
F18	HPMCAS-LG	PCM (5%) + HPMCAS-LG (95%) + Methyl paraben (15%) + Magnesium stearate (5%)	38.4 × 38.4 × 38.4	-
Polymer ratio				
F19	HPMC (5%)	Levetiracetam (23.4%) + Kollidon SR (25.9%) + SiO_2_ (10%) + HPMC (Metolose 90SH; Shin-Etsu Chemical Co., Ltd., Tokyo, Japan) (5%) + Water (35.7%)	35.4 × 35.4 × 35.4	-
F20	HPMC (10%)	Levetiracetam (23.4%) + Kollidon SR (20.9%) + SiO_2_ (10%) + HPMC (Metolose 90SH) (10%) + Water (35.7%)	30.6 × 30.6 × 30.6	-
F21	HPMC (15%)	Levetiracetam (23.4%) + Kollidon SR (15.9%) + SiO_2_ (10%) + HPMC (Metolose 90SH) (15%) + Water (35.7%)	35.5 × 35.5 × 35.5	-

Formulation compositions were adapted from the published literature (see corresponding references): F1–F3 [51], F4–F5 [52], F6–F7 [53], F8–F9 [54], F10–F13 [55], F14 [56], F15 [57], F16–F18 [58], and F19–F21 [59]. *n* = number of repeating units modeled for each excipient oligomer. For computational feasibility, large polymers were represented by shorter oligomer chains. Representative oligomers were constructed as follows: Kollicoat^®^ IR (*n* = 6); Kollidon^®^ SR (PVP/VA + EC)—oligomer block (PVP/VA (6), ethyl cellulose (10)); HPMCAS (HG/MG/LG) and ethyl cellulose: constructed according to given molecular weights; Polycaprolactone (PCL) (*n* = 10); Eudragit^®^ EPO (*n* = 6, dimethylaminoethyl methacrylate backbone); Ethyl cellulose (*n* = 10); HPMC K4M (*n* = 15); HPMC (Metolose^®^ 90SH) (*n* = 5); PEO (MW ≈ 300,000) (*n* = 300).

**Table 3:** Interaction parameter (χ) and mixing energy (E_mix_) values for various API–excipient systems calculated at reported HME processing temperatures, along with predicted miscibility from in silico analysis and experimentally reported printability.

Formulation Code	In Silico Miscibility			Experimental Reported Printability
	χ	E_mix_	Miscibility	
F1	−5.11	−3.03	Miscible	Yes
F2	26.67	20.57	Immiscible	No
F3	5.00	2.96	Immiscible	No
F4	−7.95	−6.21	Miscible	Yes
F5	13.16	10.28	Immiscible	No
F6	−13.94	−8.25	Miscible	Yes
F7	16.34	9.68	Immiscible	No
F8	0.20	0.16	Immiscible	No
F9	−12.99	−10.79	Miscible	Yes
F10	15.72	13.84	Miscible	No
F11	14.34	8.49	Immiscible	No
F12	2.50	2.26	Immiscible	No
F13	20.41	18.38	Immiscible	No
F14	−26.48	−15.68	Miscible	Yes
F15	−11.90	−10.25	Miscible	Yes
F16	8.02	7.62	Immiscible	No
F17	17.89	15.40	Immiscible	No
F18	−0.02	−0.01	Immiscible	No
F19	−5.85	−4.75	Miscible	Yes
F20	13.95	8.26	Immiscible	No
F21	−60.70	−35.94	Miscible	Yes
F22	7.74	6.97	Immiscible	No
F23	0.79	0.71	Not miscible	No
F24	0.12	0.11	Not miscible	No
F25	0.08	0.07	Not miscible	No
F26	21.79	17.89	Not miscible	No
F27	15.25	12.22	Not miscible	No
F28	34.86	27.93	Not miscible	No
F29	11.27	8.80	Not miscible	No
F30	−39.22	−38.43	Miscible	yes

**Table 4:** Correlation between calculated cohesive energy density (CED) and the in-vitro drug release profiles (T_80_, % release at 10 h, T_50_) for various 3D-printed formulations, demonstrating the influence of polymer type, grade, and concentration.

Formulation Code	Formulation Composition	T80 (Min)	CED J/cm^3^ (10^8^)
Dissolution prediction: general trends across polymers			
F1	Kollicoat IR (92%)	30	16.3
F2	Kollicoat IR (77%)	120	54
F3	Kollicoat IR (62%)	300	120
F4	HPMC (30%)	40	33.6
F5	HPMC (50%)	120	50
F6	PCL (90%)	100	94.4
F7	PCL (85%)	160	172
F8	Kollidon^®^ VA 64 (40%) +PCL (45%)	600	166
F9	Kollidon^®^ VA 64 (30%) +PCL (55%)	1500	259
F10	5-ASA + EPO (46.75%)	15	69.9
F11	Theophylline (12.5%) + Eudragit EPO (46.75%)	20	79.4
F12	Captopril (12.5%) + Eudragit EPO (46.75%)	25	58.8
F13	Prednisolone (12.5%) + Eudragit EPO (46.75%)	25	57.4
F14	HPMCAS-HG (60%)	50	128
F15	HPMCAS-HG (40%)	240	31.8
Formulation Code	Formulation Composition	% release at 10 h	CED J/cm^3^ (10^8^)
Polymer grade-dependent trends in dissolution prediction			
F16	HPMCAS-LG (95%)	95	63.9
F17	HPMCAS-MG (95%)	85	69
F18	HPMCAS-HG (95%)	30	95.4
Formulation Code	Formulation Composition	T50 (min)	CED J/cm^3^ (10^8^)
Polymer concentration-dependent trends in dissolution prediction			
F19	HPMC (5%)	240	21.4
F20	HPMC (10%)	330	28.9
F21	HPMC (15%)	480	33.8

## Data Availability

The data presented in this study are available on request from the corresponding author.
